# Safety and Tolerability of Edoxaban in Filipino Patients With Non-valvular Atrial Fibrillation: A Pilot Post-marketing Surveillance Study

**DOI:** 10.7759/cureus.104713

**Published:** 2026-03-05

**Authors:** Alisa P Bernan, Augusto Gabriel V Santos

**Affiliations:** 1 Internal Medicine, Davao Doctors Hospital, Davao, PHL; 2 Cardiology, A. Menarini Philippines, Inc., Taguig, PHL

**Keywords:** atrial fibrillation, bleeding, edoxaban, philippines, real-world evidence, safety

## Abstract

Background: Atrial fibrillation (AF) is a significant driver of cardiovascular mortality and stroke. While the safety and efficacy of the direct oral anticoagulant (DOAC) edoxaban are well-established in global trials, there is limited real-world evidence within the Filipino population. This study evaluated the safety and bleeding rates of edoxaban in routine clinical practice among Filipino patients with non-valvular atrial fibrillation (NVAF). The safety profile of edoxaban is of particular interest in the context of the "East Asian Paradox", where Asian patients exhibit a higher risk of bleeding complications on traditional anticoagulants compared to Western populations.

Methods: This was a multicenter, prospective, observational post-marketing surveillance study conducted at six sites in the Philippines, namely, Davao Doctors Hospital, St. Luke's Medical Center (Quezon City and Global City), Makati Medical Center, the Philippine General Hospital, and the Philippine Heart Center, between March 2022 and December 2024. Adult patients (≥21 years) with NVAF and a CHA2DS2-VASc (congestive heart failure, hypertension, age ≥75 years (doubled), diabetes mellitus, stroke/transient ischemic attack (TIA)/thromboembolism (doubled), vascular disease, age 65-74 years, and sex category (female)) score of ≥1 were prescribed edoxaban (30 mg or 60 mg daily) at the clinician's discretion. Patients were followed for 180 days. The primary endpoints were adverse events (AEs), adverse drug reactions (ADRs), and major bleeding.

Results: A total of 72 patients were enrolled; however, one patient (1.4%) did not receive the treatment and was excluded, resulting in a safety analysis population of 71 (mean age: 68.5±10.1 years). The majority of participants were female (50.7%; n=36). Comorbidities were highly prevalent, including hypertension (100%; n=71), coronary artery disease (25.4%; n=18), and heart failure (23.9%; n=17). In this cohort, 35.2% (n=25) were prescribed the 30 mg dose, while 64.8% (n=46) received the 60 mg dose. Over the 180-day observation period, no major bleeding, as well as bleeding requiring medical intervention but not meeting the major criteria, occurred (0%; 95% CI: 0-4.2%). The overall incidence of AEs was low (7%; n=5). One fatal serious AE (acute myocardial infarction) was reported but was determined to be unrelated to the study drug.

Conclusion: In this real-world Filipino cohort, edoxaban demonstrated a favorable safety profile with zero major bleeding events over six months of therapy. Despite a high-risk profile and significant comorbidities, these findings align with global phase III and post-marketing data, supporting edoxaban as a safe anticoagulation option for Filipino patients with NVAF.

## Introduction

Atrial fibrillation (AF) is one of the most significant health issues worldwide, with risk factors for stroke, heart failure, and cardiovascular disease-related death [1]. In the Philippines and Asia, AF is rising with an aging population and increasing burden of cardiovascular risk factors (hypertension and diabetes) [2,3]. In Asia alone, the prevalence of AF ranges from 0.3% to 3.5% within community settings and escalates to 2.8-15.8% in hospital populations [4]. For non-valvular atrial fibrillation (NVAF), oral anticoagulation is considered the standard of care for stroke prevention.

Traditionally, vitamin K antagonists (VKAs) like warfarin have been utilized as the first line of care; however, due to most having a narrow therapeutic index, food-drug interactions, and frequent monitoring requirements, their use is frequently restricted. Direct oral anticoagulants (DOACs) have now become the safer option of choice, with pharmacokinetics that is predictable and a non-inferior effect to warfarin. In addition, different landmark trials have shown the superiority of DOACs over warfarin in the prevention of major bleeding events [4].

Direct factor Xa inhibitors include edoxaban, which showed non-inferiority in stroke prevention and systemic embolism compared to warfarin at very low major bleeding and major cardiovascular death rates in the pivotal ENGAGE AF-TIMI 48 (Effective aNticoaGulation with factor Xa next Generation in Atrial Fibrillation-Thrombolysis in Myocardial Infarction study 48) trial. The strict inclusion criteria typically used in clinical trials do not accurately represent the population of patients that may be encountered in practice. Moreover, ethnic variations in coagulation profiles and bleeding risks, specifically, the so-called "East Asian Paradox", when Asian patients can possess a more significant risk of intracranial hemorrhage on warfarin, require local data [5]. The "East Asian Paradox" describes a unique clinical phenomenon in which patients of East Asian ethnicity demonstrate a higher susceptibility to bleeding, specifically intracranial hemorrhage, when treated with VKAs, despite having a lower overall risk of thromboembolism. This population often requires lower dose intensities of anticoagulation to balance safety and efficacy, highlighting the need for local real-world data on newer agents like edoxaban.

Currently, real-world evidence specifically characterizing the safety profile of edoxaban in the Filipino population is limited. This post-marketing surveillance study aimed to evaluate the safety, tolerability, and bleeding outcomes of edoxaban in Filipino patients with NVAF treated in routine clinical settings.

## Materials and methods

Study design and setting

This was a prospective, multicenter, observational post-marketing surveillance study conducted across six participating institutions in the Philippines: Davao Doctors Hospital, St. Luke's Medical Center (Quezon City and Global City), Makati Medical Center, the Philippine General Hospital, and the Philippine Heart Center. The study was implemented from March 31, 2022, to December 4, 2024, after obtaining approval from the Single Joint Research Ethics Board of the Department of Health (approval number: SJREB-2022-90). The observation period spanned 180 days on a per-patient basis and consisted of four scheduled clinical visits: visit 1 (baseline), visit 2 (day 30), visit 3 (day 90), and visit 4 (day 180). The research was conducted in accordance with the Declaration of Helsinki [6] and established local regulatory guidelines for clinical studies.

Study population

Inclusion criteria consisted of male and female patients aged 21 years or older with a diagnosis of NVAF and a CHA2DS2-VASc (congestive heart failure, hypertension, age ≥75 years (doubled), diabetes mellitus, stroke/transient ischemic attack (TIA)/thromboembolism (doubled), vascular disease, age 65-74 years, and sex category (female)) score ≥1. The CHA2DS2-VASc score, a validated clinical tool used to estimate thromboembolic stroke risk, was calculated by assigning one point each for congestive heart failure, hypertension, age 65-74 years, diabetes mellitus, vascular disease, and female sex and two points each for age ≥75 years and a history of stroke, TIA, or thromboembolism [7].

Higher scores correspond to an increased risk of stroke, indicating a greater need for anticoagulation. Patients were either treatment-naive or transitioning to edoxaban from another anticoagulant, such as a VKA or a different DOAC. Patients were excluded if they had hypersensitivity to edoxaban, hepatic disease associated with coagulopathy, end-stage renal disease (defined as a creatinine clearance (CrCl) <15 mL/min), uncontrolled active bleeding, or severe hypertension.

Treatment

Edoxaban was prescribed at the clinical discretion of the treating physician in accordance with local treatment guidelines and the approved product label. The standard recommended dose was 60 mg once daily. A reduced dose of 30 mg once daily was administered to patients meeting one or more of the following criteria: moderate or severe renal impairment (CrCl 15-50 mL/min), body weight ≤60 kg, or concomitant use of potent P-glycoprotein (P-gp) inhibitors.

Renal function was assessed via the estimation of CrCl, which was used to determine the appropriate edoxaban dosing (60 mg vs. 30 mg). CrCl was calculated using the Cockcroft-Gault equation [8], which incorporates the patient's age, body weight, and serum creatinine levels. For female patients, the resulting value was multiplied by 0.85 to account for lower muscle mass.

Endpoints and assessments

The primary objective of the study was to assess safety. The safety endpoints were the incidence of adverse events (AEs), adverse drug reactions (ADRs), serious adverse events (SAEs), and, specifically, major bleeding episodes. Major bleeding was defined according to the International Society on Thrombosis and Haemostasis (ISTH) criteria [9]. An event was classified as major if it was clinically overt and met at least one of the following: (1) fatal bleeding; (2) symptomatic bleeding in a critical area or organ, such as intracranial, intraspinal, intraocular, retroperitoneal, intra-articular, pericardial, or intramuscular with compartment syndrome; or (3) bleeding causing a fall in hemoglobin level of 2 g/dL (1.24 mmol/L) or more or leading to transfusion of two or more units of whole blood or red cells.

Patients were evaluated at each scheduled visit for AEs through medical interviews, physical examinations, and the assessment of vital signs.

Statistical analysis

Baseline characteristics and safety outcomes were summarized and analyzed using descriptive statistics. Continuous variables, such as age and weight, were presented as means with standard deviations (SD). Categorical variables, including sex, comorbidities, and the incidence of AEs, were expressed using frequencies and percentages. The safety analysis population included all enrolled patients who received at least one dose of the study medication. Of the 72 patients initially enrolled, one did not receive treatment and was excluded, resulting in a final safety population of 71 participants.

## Results

Patient disposition and baseline characteristics

A total of 72 patients were enrolled in the study. However, one patient (1.4%) did not receive the study medication and was consequently excluded from the analysis. Therefore, 71 patients constituted the final safety analysis population. The study demonstrated high retention, with 90.1% (n=64/71) of participants completing the full 180-day follow-up. Safety analysis was performed on the intent-to-treat population (N=71), with data censored at the time of last contact for discontinued patients.

The baseline demographic and clinical characteristics are summarized in Table 1. The mean age of the safety population was 68.5±10.1 years. Contrary to early estimates, the majority of the participants were female (50.7%; n=36), while males represented 49.3% (n=35) of the cohort. The population exhibited a high prevalence of cardiovascular comorbidities: hypertension was present in 100% (n=71) of patients, followed by coronary artery disease in 25.4% (n=18) and congestive heart failure in 23.9% (n=17).

**Table 1:** Baseline clinical and demographic characteristics of the safety analysis population (N=71) CHA2DS2-VASc scores were used to stratify stroke risk, with high risk defined as a score ≥2 for males and ≥3 for females. CHA2DS2-VASc: congestive heart failure, hypertension, age ≥75 years (doubled), diabetes mellitus, stroke/transient ischemic attack (TIA)/thromboembolism (doubled), vascular disease, age 65-74 years, and sex category (female)

Characteristic	Frequency (n)	Percentage (%)
Age, years (mean±SD)	68.5±10.1	-
Gender		
Female	36	50.7%
Male	35	49.3%
Body weight, kg (mean±SD)	64.9±11.2	-
Creatinine clearance, mL/min (mean±SD)	66.8±26.3	-
Dosing regimen		
Edoxaban 60 mg once daily	46	64.8%
Edoxaban 30 mg once daily	25	35.2%
Comorbidities		
Hypertension	71	100%
Diabetes mellitus	28	39.4%
Coronary artery disease	18	25.4%
Congestive heart failure	17	23.9%
Concomitant antiplatelet use (aspirin/clopidogrel)	14	19.7%
Stroke risk profile (CHA2DS2-VASc)		
High risk (score ≥2 for males, ≥3 for females)	63	88.7%
Intermediate risk (score 1 for males, 2 for females)	8	11.3%

Stroke risk profile

Per the inclusion criteria, all patients had a CHA2DS2-VASc score ≥1. The cohort displayed a high-risk stroke profile, with 90.7% (n=39/43) of female patients and 85.7% (n=24/28) of male patients classified as high risk. These findings underscore a significant clinical requirement for effective anticoagulation within this specific real-world population.

Dosing patterns

Prescribing patterns reflected clinical adherence to dose-reduction criteria based on renal function and body weight. In this cohort, the standard 60 mg once-daily dose was utilized in 64.8% (n=46) of patients. The reduced 30 mg once-daily dose was administered to the remaining 35.2% (n=25) of the population. This dosing distribution is consistent with the management of an elderly and often frail Filipino population with high rates of renal impairment or low body weight.

Safety outcomes and AEs

Overall, AEs were infrequent during the observation period. A total of 7.04% (n=5) of patients reported at least one AE over the six-month period. These events included bradycardia, hemorrhoids, hyperchlorhydria, and impaired fasting glucose. The majority of these events were mild in severity and were assessed by investigators as "not related" or "unlikely related" to edoxaban. The summary of safety outcomes is presented in Table 2.

**Table 2:** Summary of adverse events (N=71) ISTH: International Society on Thrombosis and Haemostasis

System organ class (SOC)/preferred term (PT)	Number of events	Percentage of population (n/N)
Cardiac disorders	2	2.8%
Bradycardia	1	1.4%
Acute myocardial infarction	1	1.4%
Gastrointestinal disorders	2	2.8%
Hyperchlorhydria	1	1.4%
Hemorrhoids	1	1.4%
Metabolism and nutrition disorders	1	1.4%
Impaired fasting glucose	1	1.4%
Total adverse events	5	7.04%
Total major bleeding (ISTH criteria)	0	0%

Bleeding events

There were no major bleeding events (0%; n=0) reported within the study cohort. Specifically, there were no instances of intracranial hemorrhage, gastrointestinal bleeding requiring transfusion, or fatal bleeding events. One subject (Subject 600009) experienced a mild AE of blood-streaked stool related to pre-existing hemorrhoidal disease. This event was determined to be non-serious and resolved without the need to discontinue the study drug.

SAEs and mortality

One death occurred during the study, resulting in a mortality rate of 1.4% (n=1). The case involved a 65-year-old man (Subject 400011) with a history of hypertension and chronic obstructive pulmonary disease (COPD) who suffered a new-onset acute myocardial infarction (AMI) on day 45. The investigator concluded that this SAE was unrelated to edoxaban and was instead linked to the patient's underlying cardiovascular disease. No other SAEs were reported during the study.

## Discussion

Being a post-marketing surveillance study, this is the first dedicated real-world evidence of the safety profile of edoxaban in Filipino patients with NVAF. These results are very encouraging, with a good tolerability profile, and, more importantly, there was no report of major bleeding.

The absence of major bleeding observed in this cohort is a significant finding. For context, in the global ENGAGE AF-TIMI 48 trial, the annualized rate of major bleeding was 2.75% for high-dose edoxaban [5]. Similarly, the ETNA-AF-Japan (Edoxaban Treatment in routiNe clinical prActice in patients with nonvalvular Atrial Fibrillation) post-marketing study reported a major bleeding incidence of approximately 1.04% per year [10]. This finding suggests that the safety benefits of edoxaban are maintained when compared with existing real-world evidence and that there is a favorable clinical acceptance in the Filipino setting. The physicians also correctly followed the 30 mg vs. 60 mg label instructions.

Another significant observation was that a subset of patients in our study were receiving concomitant antiplatelet therapy (aspirin or clopidogrel) for coronary artery disease. The concomitant use of antiplatelets is a known risk factor that significantly increases bleeding risk in anticoagulated patients. In a post hoc analysis of the ENGAGE AF-TIMI 48 trial, it was shown that patients on a high-dose edoxaban regimen (HDER) plus single antiplatelet therapy (SAPT) had a major bleeding rate of 3.55% per year, compared to 2.04% per year in those not on SAPT. Furthermore, major bleeding was assessed to be 1.5 times higher in patients on combination therapy [11]. The fact that these "dual-therapy" patients did not experience major bleeding further validates the safety profile of edoxaban in higher-risk scenarios. Despite 19.7% of the cohort receiving concomitant antiplatelet therapy, no major bleeding events were observed, further supporting the safety of edoxaban in high-risk Filipino patients. Edoxaban demonstrated a favorable safety profile and was well-tolerated in this pilot cohort.

The dosing regimens (65% on 60 mg/35% on 30 mg) described suggest that Filipino treating physicians were able to adequately follow proper use of the drug following the dose-reduction criteria. This strict adherence to the recommendations on the label could have contributed to the low bleeding rates. The proper use of the "30 mg" dose offers a decreased risk of bleeding in patients with a higher propensity to experience a major bleeding episode (use of P-gp inhibitors, low body weight, renal dysfunction) such as that seen in the ENGAGE AF-TIMI 48 study [12,13].

Limitations

This study has limitations inherent to its observational design, including the absence of a control arm. The sample size (n=71) and follow-up duration (six months) may not be sufficient to detect very rare AEs. However, as a real-world snapshot of clinical practice, it provides valuable local validation of global trial data.

## Conclusions

Edoxaban had a good safety profile in Filipino patients with NVAF, with an overall low incidence of AE and zero major bleeding over a six-month follow-up. Edoxaban demonstrated a favorable safety profile and was well-tolerated in this pilot cohort. Our findings corroborated the use of edoxaban as a safe DOAC for stroke prevention in a Philippine population.
