# Correction: Safety and Tolerability of Edoxaban in Filipino Patients With Non-valvular Atrial Fibrillation: A Pilot Post-marketing Surveillance Study

**DOI:** 10.7759/cureus.c418

**Published:** 2026-03-26

**Authors:** Alisa P Bernan, Augusto Gabriel V Santos

**Affiliations:** 1 Internal Medicine, Davao Doctors Hospital, Davao, PHL; 2 Cardiology, A. Menarini Philippines, Inc., Taguig, PHL

This article has been corrected to address the erroneous inclusion of preliminary data that does not match the finalized, correct data displayed in Table 1. The following revision has been made in the 'Dosing Patterns' paragraph of the Results section:

- Original: "In this cohort, the lower 30 mg once-daily dose was more frequently utilized, prescribed to 54.9% (n=39) of patients. The standard 60 mg once-daily dose was administered to the remaining 45.1% (n=32) of the population."
- Revised: "In this cohort, the standard 60 mg once-daily dose was utilized in 64.8% (n=46) of patients. The reduced 30 mg once-daily dose was administered to the remaining 35.2% (n=25) of the population."

